# From Stigma to Strength: Tracking Post-Traumatic Growth in Older Sexual Minorities

**DOI:** 10.1093/geroni/igaf122.2402

**Published:** 2025-12-31

**Authors:** Michael Vale

**Affiliations:** Sacred Heart University, Fairfield, Connecticut, United States

## Abstract

Post-traumatic growth (PTG) refers to the personal development that arises from overcoming adversity and has historically been overlooked in older sexual minorities (e.g., lesbian, gay, bisexual individuals). Throughout their lives, older sexual minorities have faced heightened vulnerability to marginalization (e.g., discrimination), which has been linked to worse health outcomes. However, over time, they may develop a sense of adaptation to these stigmatizing experiences, establishing a potential source of resilience (i.e., PTG). Yet, no existing scales specifically capture PTG in queer populations. This study aimed to (1) establish a PTG measure for sexual minorities and (2) examine PTG’s correlates in younger (N = 107, ages 18–39) and middle-aged/older (N = 216, ages 40–90) sexual minorities. Exploratory factor analysis supported the hypothesized five-factor structure with strong internal consistency (α = .88). Although PTG was not correlated with age (p = .21), it was associated with microaggressions (r = .16, p < .05), discrimination (r = .24, p < .001), and victimization (r = .24, p < .001) across age groups. PTG was also associated with other adaptive psychosocial resources, such as self-compassion (r= .20, p<.05), active coping (r= .21, p<.05), religious coping (r= .36, p<.001), and positive reframing (r= .33, p<.001), but only among the older sexual minorities. These results highlight that PTG may play a unique role in promoting well-being for this health disparity group and serve as a catalyst for future research on PTG in this population. Specifically, researchers should explore interventions that harness PTG to enhance well-being.

